# Nurses, midwives and students' reports of effective dedicated education units in five European countries: A qualitative study

**DOI:** 10.1002/nop2.2210

**Published:** 2024-07-03

**Authors:** Sara Pedregosa, Adelaida Zabalegui, Núria Fabrellas, Ester Risco, Mariana Pereira, Ewa Dmoch‐Gajzlerska, Fisun Şenuzun, Sandra Martin

**Affiliations:** ^1^ Facultat d'Infermeria i Fisioteràpia Universitat de Lleida Igualada Spain; ^2^ Hospital Clínic de Barcelona Barcelona Spain; ^3^ Faculty of Medicine and Health Sciences University of Barcelona Barcelona Spain; ^4^ Nursing Research Group, Hospital Universitari Parc taulí, Institut d’Investigació i Innovació Parc Taulí (I3PT‐CERCA) Universitat Autoonoma de Barcelona Sabadell Spain; ^5^ Instituto Politécnico de Setúbal Setúbal Portugal; ^6^ Medical University of Warsaw Warszawa Poland; ^7^ Ege University Faculty of Nursing Internal Medicine Nursing Izmir Turkey; ^8^ Center of Expertise Health Innovation at UC Leuven‐Limburg Diepenbeek Belgium

**Keywords:** nurse roles, nursing students, practice‐based learning, qualitative approaches

## Abstract

**Aim:**

To investigate nursing/midwifery students, Clinical Mentors, Link Teachers and Head Nurses experiences within “Dedicated Education Unit” model in 6 European clinical placements and analyse the necessary elements for a powerful clinical learning environment.

**Design:**

A multi‐country, phenomenological, qualitative study.

**Methods:**

Focus group interviews were performed to identify the personal and organizational factors of importance for students and nurses/midwives.

**Results:**

Data analysis produced 4 main themes (1) Clinical placement organization, (2) students' clinical knowledge and skill acquisition, (3) students, and nurses/midwives' experiences within the DEU model and (4) factors for creating an effective learning environment.

**Conclusions:**

A close educational‐service collaboration, a realistic clinical placement planning, a focus on student learning process and an investment in professionals’ education and development among others, are elements to set up a powerful clinical learning environment.

**Implications for the profession:**

It is considered advisable and urgent to improve the working conditions of nurses/midwives and the learning environments of students as a strategy to alleviate the global shortage of nurses and respond to the increasingly demanding health needs of the population.

**Impact:**

Due to the close relationship between students’ learning and features of the clinical environment nurse educators seek innovative models which allow students to manage patient care and their transition to professional practice. To implement new learning strategies, identifying students, nurses and midwives perceptions and suggestions is a powerful information to evaluate implementation process and outcomes.

**Public Contribution:**

Our findings could help academic and clinical managers to meet the human and organizational requirements to create a successful learning environment in every student placement.

## 1 INTRODUCTION

Clinical learning environment is vital to offer students a place to develop knowledge, critical thinking and communication and problem‐solving skills (Arkan et al., 2018; Jayasekara et al., 2018). The quality of the learning environment depends on student learning opportunities, the collaboration between academic and clinical services and the relationship among students, health professionals and university faculty members (Arkan et al., 2018). However, the literature describes differences in the quality of placements and in students' experiences and shows that not all clinical sites are optimal learning environments (Arkan et al., 2018; Immonen et al., 2019). Unfavourable environments for students due to organizational weaknesses, a poor relationship between students and supervisors and negative attitudes and behaviours from these professionals have been described. In addition, the distance between educational and health institutions, the lack of supervision and feedback to the student, the lack of clinical learning opportunities, the lack of the student's sense of belonging, the gap between theory and practice or situations that produce a lack of student confidence in expressing doubts have been showed as limiting factors for an optimal learning environment (Cant et al., 2021; Courtney‐Pratt et al., 2018; Immonen et al., 2019; O'Brien et al., 2019; Panda et al., 2021). According to the perspectives of students and professionals, the qualities of an effective clinical environment would be a learning atmosphere based on teamwork, good motivation and communication with the health team, management with democratic leadership, a good relationship with the teaching and clinical nurses, high‐quality care and measures that provide opportunities for professional development, among others (Arkan et al., 2018; Pitkänen et al., 2018). The expanded traditional model (TM) of nursing/midwifery students' placement typically involves a group of 8–10 undergraduate students working with different nurses/midwives while being supervised by a clinical teacher. Nurse educators seek innovative learning models that allow students to build the knowledge and skills to safely and effectively manage patient care and their transition to practice (Jayasekara et al., 2018). On the other hand, it is recognized that, in certain organizations, nurses/midwives have to provide quality care in the midst of nursing shortage, a high workload, and a large amount of time allocated to administrative tasks and obligations related to evaluating the care quality. At the same time, these nurses/midwives have to instruct and supervise students and ensure that students have the knowledge, skills and competencies necessary for autonomous work (Chan et al., 2019; Wu et al., 2018).

Due to these realities, education service managers need to find alternative methods that meet the needs of the current and future generations of the nursing workforce. The Dedicated Education Unit (DEU) model has emerged as an alternative to standard models for being beneficial to improve the students' clinical experiences and professional growth (Pedregosa et al., 2020). In the last few decades, the model has spread to the United States, New Zealand and Europe as it improves students' clinical experiences and their professional development (Clarke et al., 2020). A recent review on the effect of the DEU on students' clinical experience summarizes that the DEU improves student knowledge, competence, clinical self‐efficacy, confidence, teamwork and collaboration skills, and produces greater satisfaction with their clinical experience (Musallam et al., 2021). Also, clinical nurses who participated in studies evaluating the DEU reported high levels of satisfaction, effort and support from the rest of the team, and knowledge and tools that improved their teaching skills and career opportunities (Glynn et al., 2019; Jones et al., 2017; Marcellus et al., 2021). Additionally, the DEU has successfully bridged the education–practice gap by extending the nurse mentor role in instructing and supervising students' clinical skills in the clinical setting and the nurse teacher role in meeting academic curriculum objectives, ensuring student knowledge acquisition and coaching clinical nurses in their teaching activities (Huston et al., 2018; Jayasekara et al., 2018; Pedregosa et al., 2020; Williamson et al., 2020). The main characteristics of the DEU, as outlined by Moscato et al. (2013), are shown in the following box:

**Table:** 

Committed partnership between academic and healthcare organizations. Extended students' clinical placement period, from 6 to 12 weeks. A staff nurse/midwife acts as a mentor who guides, instructs and supervises undergraduate students in clinical placement in a one‐to‐one relationship. Termed clinical mentor in our study. A faculty nurse/midwife acts as a link between academic service institutions and is responsible for mentor–student partnership coordination, mentor coaching and learning‐teaching process evaluation. In our study, a faculty or clinical nurse/midwife was termed a link teacher. The ward manager is involved in the teaching‐learning process and creates conditions for a ward‐learning culture. Termed head nurse in our study. A trainers' course is provided to mentors with pedagogical education, skills and support necessary to sustain student learning. Meetings between mentors, teachers, head nurses and students are scheduled to enhance feedback and communication, evaluate the students' learning needs and agree on the teaching/learning process.

### 1.1 Aim of the research

**FIGURE 1:**
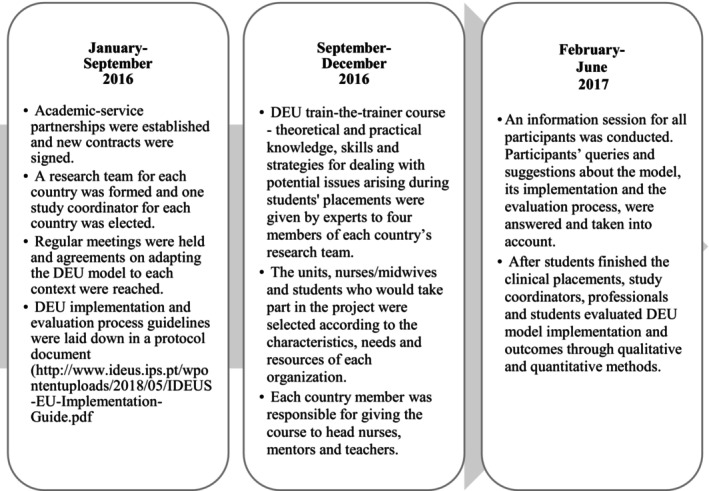
DEU model pilot implementation and evaluation process.

The aim of the present study was to describe the students and nurses/midwives' experiences within the DEU and their suggestions for an optimal clinical learning environment. This is a key factor in the evaluation of the quality of educational programmes and a powerful tool to find the elements in an effective clinical learning environment. This qualitative study is part of a larger study exploring the implementation and the results of DEU in five European countries. No studies have described the teaching‐learning process within the DEU in European countries' clinical placements from the perspective of professionals and students. Our process to implement and evaluate the DEU in each country's student placement is shown in Figure 1.

## 2 METHODS

### 2.1 Design

This is a qualitative study, in that we are attempting to understand the meanings of the lived experience of the students and nurses/midwives within DEU through the discussions and reflections of a group. The paradigmatic perspective is in the framework of social constructivism, focused on the collective generation of meaning, in this case specifically via language and the social interaction in the group (Berger et al., 1968). The method of investigation used was the grounded theory design. We considered this method to be most appropriate for getting closer to antecedent‐free subjective processes because it allows us to use the frame of reference of the agents actually involved (Strauss & Corbin, 1997).

### 2.2 Research emplacement

This study was carried out in 6 higher educational and 6 healthcare institutions from 5 European countries that had implemented the DEU: Belgium, Portugal, Poland, Spain and Turkey. The characteristics of clinical model in most of these placements before DEU were a lesser clinical period (from 22 days to 8 weeks), the clinical nurse instructs 2–3 students simultaneously, clinical nurses are not trained to instruct students and/or the teacher role does not exist.

### 2.3 Study participants

A convenience sample was obtained by sending an e‐mail to the 40 nursing/midwifery students who had completed their clinical experience in the DEU and to the 39 mentors/teachers who had trained or supervised these students to emplace them for focus group interviews. Mentors were registered nurses/midwives with at least 1 year of experience on the ward. Teachers were faculty or clinical nurses with past and/or future long‐lasting connections with the ward (long‐term contracts), who knew the patient population and the healthcare institution, held master's degrees and had pedagogical competency (competencies to plan, implement and evaluate students' learning outcomes provided by the educational institution). Students were nursing/midwifery students who had done their last placement in the DEU. Students' demographic data are described in Table 1.

**TABLE 1:** Students' demographic data.

*Age of the students*		
Mean	21.5	
Range	20–26	
**Previous clinical practice**	***n***	**%**
Yes	38	95%
No	2	5%
*Total weeks of previous clinical practices*		
0 week	2	5%
0–20 weeks	13	25%
20–30 weeks	7	17.5%
30–40 weeks	13	32.5%
>30 weeks	5	12.5%
*Dedicated education units in which students completed clinical practices*		
Critical Care Unit	18	45%
Obstetrics and Gynaecology Unit	8	20%
Emergency Room	3	7.5%
Traumatology Unit	3	7.5%
Paediatric Unit	2	5%
Maternity and delivery room	2	5%
Operating Room	4	10%

### 2.4 Data collection

Participants received both written and oral information on study aims and methodology, and informed consent was obtained from each participant before the interviews. The recruitment stage, the informed consent process and data collection were carried out by professionals who do not present a relationship dependence with the participants. Also, authors, unacquainted with the study participants moderated the focus groups using a semi‐structured interview guide (Table 2). One observer took notes about participants' interactions and non‐verbal communication. Students were divided into six groups of a maximum of 14 students, and nurses/midwives were arranged into seven groups of a maximum of 6 participants. Focus group duration was 60–90 min and were located separate to the usual participants' placements. Each interview was recorded and transcribed in the country's language.

**TABLE 2:** Questions to guide focus group discussion.

Main question	Specific questions
1 What were the biggest differences between the DEU and traditional placement in your view?	Concerning the duration of clinical placements? Concerning the collaboration between educational organization and healthcare facility? Concerning the one‐to‐one relationship with clinical mentors? Other relevant differences in common?
2 What was the one‐to‐one relationship with the clinical mentor like in the DEU?	Concerning trust? Concerning independence in organizing care (connected/disconnected tasks)? Other relevant experiences in common?
3 How did you find the presence and collaboration of the link teacher in the DEU?	Did you experience a difference in learning climate compared with other placements? If so, what differences did you notice? What were the benefits of a placement in the DEU in your view? Could you make suggestions to improve the learning experience in the DEU?
4 What would you change in the placement?	
*The following questions were added to clinical mentor, link teacher and head nurse focus group interviews*	
5 Did you experience any difference in learning climate for students compared with traditional placements? If so, what differences did you notice?	
6 What were the benefits of the DEU in your view?	
7 Could you make suggestions to improve the learning experience in the DEU?	

Abbreviation: DEU, Dedicated Education Unit.

### 2.5 Data analysis

Each transcription was read independently by all researchers in the analysis and then was analysed alongside field notes taken by the observer. The researchers were logistically challenged to conduct the analysis together, categorize keywords or phrases and organize them into standard themes throughout the study. They applied template analysis to code the verbatim transcripts from the focus groups (King, 2004). The first template was based on the interview guide and contained four main themes. Five researchers (REDACTED) familiarized themselves with the data from focus groups and started coding the data, based on a combined inductive and deductive approach. Finally, categories were compared and contrasted, and the researchers searched for contradictions until an agreement was reached. All authors provided feedback on the write‐up presentation of findings, according to the appropriate revisions of the paper. Computer software (ATLAS‐ti version 8.2.1) was used for exploration, management and evaluation of data. Standards of quality and scientific rigour were followed (Cohen & Crabtree, 2006); (a) credibility: data were verified by the informants ensuring isomorphism between the data collected and what they said; (b) transferability: hired trained, skilled and qualified professionals translated raw data into English and back‐translated to preserve maximum accuracy of participants' expressions within the context, safeguarding study validity and cross‐language trustworthiness; (c) dependence: researchers checked the process of the data analysis and (d) confirmability: verbatim transcription draft kept safe for verification.

### 2.6 Ethical considerations

The Ethics and Research Committee at (REDACTED) granted study approval (number: HCB2017/0053) Participants received both written and oral information on study aims and methodology, and informed consent was obtained from each participant before the interviews. Participants' personal information was collected by code to ensure confidentiality. Professionals and students were free to contribute to the study. If they chose not to participate, all the services they received at these organizations were continued and it did not affect any aspect of their learning‐working or related evaluations in any way.

## 3 RESULTS

Between February and June 2017, on completion of DEU placement (from 7 to 12 weeks), a purposive sample of 31 nursing and 2 midwifery students (rate response: 86.8%). participated in 6 focus groups. Belgian A (*n* = 7) and B (*n* = 5), Polish (*n* = 2), Portuguese (*n* = 2), Spanish (*N* = 14) and Turkish (*N* = 3). Students' average age was 21.5 years (min. 20, max. 26). Prior to the DEU, only 2 midwifery students had no previous clinical experience (the training of midwives did not require completing nursing education). Additionally, 8 head nurses/midwives, 8 teachers and 18 mentors (rate response: 86.7%). participated in 7 focus group interviews. Belgian A (*n* = 5), B (*n* = 4) and C (*N* = 5), Poland (*n* = 4), Portugal (*n* = 6), Spain (*n* = 6) and Turkey (*n* = 4). The average age of nurses and midwives was 44.4 years (min. 26, max. 64). Data analysis produced four main themes (1) placement organization, (2) students' clinical knowledge and skill acquisition, (3) students and nurses/midwives' experiences within the DEU model and (4) factors for creating an effective learning environment (Table 3). Under themes explanation we added representative comments of participants. More participants comments are attached in File S1.

**TABLE 3:** Themes and categories of the focus groups data.

Theme	Category
1. Clinical placement organization	1.1 Collaboration between educational and service institutions 1.2 Focus on the student‐learning process 1.3 Longer clinical placement is better for student learning 1.4 One‐to‐one is the best ratio for the teaching‐learning process
2. Students' clinical knowledge and ability acquisition	2.1 From theory to practice
3. Students and nurses/midwives within the Dedicated Education model	3.1 Roles and responsibilities in the teaching/learning process 3.2 Participant interaction 3.3 Students' sense of belonging to the healthcare team 3.4 Nurses and midwives' involvement in student learning
4. Participants' suggestions for creating an effective clinical learning environment	4.1 Agreement on learning–teaching process 4.2 Clinical placement early planning 4.3 Investment in nurses and midwives

### 3.1 Theme 1. Clinical placement organization

#### 3.1.1 Collaboration between educational and service institutions

Students stated that there was better collaboration between academic and healthcare institutions in the DEU versus the TM. This new partnership brought students' curricula closer to the real activity of the clinical setting. Nurses/midwives expressed appreciation for this collaboration and viewed it as a positive strategy, as it allowed everyone to work towards the same goals, updated the faculty's clinical knowledge and provided staff with effective education strategies. A mentor stated ‘Collaboration between institutions was very good (in the DEU); we all became part of the same team’.

#### 3.1.2 Focus on the student‐learning process

In the DEU, weekly meetings with students, teachers, mentors and head nurses were scheduled throughout the placement. The teaching–learning process was evaluated, and opportunities were given to raise queries and discuss needs. Students stated they had more learning opportunities in the DEU versus the TM, where students performed simple, disconnected clinical tasks and had few opportunities to perform total patient care. Students added that in the TM they had to constantly ask nurses/midwives for something to do/learn. In the DEU, mentors and teachers described a greater commitment to finding new learning opportunities. A student added: ‘The main difference (within the DEU), is a greater follow‐up. Weekly meeting (in the DEU) allows everyone to follow the whole process better’.

#### 3.1.3 Longer placement enhances student autonomy and responsibility

Most students said that longer placement is better as the student gets to know the ward, routines, staff and patients in their first 3–4 weeks of placement. Subsequently, students have enough time to gain autonomy and responsibility for the development of independent and professional care skills. Students and nurses/midwives considered that during the final weeks of the DEU placement, students were immersed inward procedures and habits, with the professional responsibilities and challenges of a staff nurse/midwife, but with supervision and support available. A student stated: ‘…It takes three to four weeks to get to know the unit, to be oriented’. ‘(After this), I can understand better how patient care is organized and the tasks and the contributions of other caregivers’.

#### 3.1.4 One‐to‐one student to mentor is the best ratio for the teaching–learning process

Participants agreed on the benefits of the one‐to‐one student–mentor relationship. All stated that this individual supervision enhanced student learning due to its orientation to students' personal learning goals and that it took students' traits or learning styles into account. In the DEU, students and mentors highlighted the benefit of the connection with a single person throughout the placement without the time‐consuming effort of building a new relationship and adapting to other learning and teaching habits. A student stated: ‘That you can work mostly with the same nurse, who is a mentor and you don't need to adjust to someone else. Not adapting every time…’.

### 3.2 Theme 2. Students' clinical knowledge and skill acquisition

#### 3.2.1 From theory to practice

In the DEU model, students could provide total patient care focused on mentor set‐tasks, including administrative tasks, organization of care delivery and cooperation with other professionals. Students stated that their critical and clinical thinking, autonomy, responsibility and self‐confidence regarding patient care had improved. Students said that they had more awareness of their own learning took greater responsibility for it and felt less pressure and anxiety and more prepared in the final assessments. Participants still noted a gap between classroom theory and practice, which produced a deep sense of confusion in students and in nurses and midwives' expectations about the clinical learning and knowledge of students. Consequently, students agreed on teacher‐led weekly seminars and written patient‐care reflections, while increased teacher presence on the ward contributed to bridging the theory–practice gap. Mentors highlighted the teachers' indispensable contribution in bringing them theoretical support and educational skills, and matching clinical practice with students' curricula. A mentor stated: ‘Teachers should give theoretical support, protect didactic matters, watch over what students should learn according to the syllabus, and mediate problems between student and mentor’.

### 3.3 Theme 3. Students and nurses/midwives within the DEU model

#### 3.3.1 Roles and responsibilities in the teaching–learning process

This study revealed the difficulties experienced by teachers and mentors in defining their roles and understanding their responsibilities in student learning in the TM. Mentors and teachers found that the DEU preparatory training and continuous feedback provided clarification on roles and responsibilities within the healthcare team, and allowed them to act according to their responsibilities. Also, the training on teaching methods and attitudes made them feel more prepared for clinical education. In the DEU, the teachers had fewer duties in students' clinical activities and could focus on providing coaching to mentors. Participants valued the teacher knowing ward routines, and staff characteristics allowed students to be more comfortable in the clinical site, have a better understanding of clinical situations and have more empathy with staff working or teaching habits. Moreover, the teacher felt a sense of belonging to the healthcare team and their greater presence on the ward during students' placement allowed them to interact with students, mentors and patients. Mentors and head nurses stated that it helped the learning process, promoted a deeper understanding of ward routines and gave them support and guidance in improving their teaching abilities and in redirecting their educational habits.

Students and mentors stressed that the mentor is a role model for student socialization, professional development and leadership. Mentors stated that their role improved their awareness of students' duties and encouraged them to express their knowledge and abilities as a professional and instructors. Some mentors stated that fruitful mentorship required student motivation, interest and willingness to learn.

Students agreed that in the DEU, the head nurse showed greater interest in the students' clinical experience, and they perceived a closer relationship than in the TM. Likewise, staff stated that the head nurses were much more aware of teaching–learning opportunities when ward duties allowed it, the head nurse actively participated in teaching about patients' healthcare problems, protocols and nursing interventions to enhance clinical learning and reduce nurses/midwives' teaching tasks. A head nurse added: ‘Thanks to individual approach to students and very clear distinction between clinical mentor and link teacher, both theoretical and practical education was much easier’.

#### 3.3.2 Participant interaction

Some students perceived a sustained lack of communication between the university and the healthcare services, and differences between university learning objectives and what mentors expected from them in clinical settings. However, professionals found improvements in academic–service's interaction and communication and this close cooperation promoted familiarity and confidence with student‐learning outcomes on the clinical ward and with student assessment criteria. Furthermore, participants stated that weekly meetings with teachers, mentors, head nurses and students allowed time for feedback. Although almost all students said they had daily or weekly feedback from their mentors, some said they occasionally missed the desired feedback from the mentor, mainly due to nurses/midwives' workload. Students described a closer relationship and greater mentor attention and that they were open to talking about their personal or clinical experiences. In the DEU, students had more trust in the mentor than in their teacher, given that the mentor was a nurse/midwife, who was always with them during placement, offered regular feedback and demonstrated knowledge of students' personal and professional traits. Also, mentor trust enabled students to care for patients more independently. A mentor stated: ‘More communication between us and with the teacher. We discuss more than before. There (in the traditional model) were a lot of different teachers, they changed a lot and we did not know them well’.

#### 3.3.3 Students' sense of belonging to the healthcare team

Students expressed a common perception regarding mentors' attitudes and teaching methods, and the inadequate support from the rest of the team had negative effects on student motivation and learning. In the TM, students avoided asking questions to the staff because they felt their practical and theoretical knowledge was being judged and criticized. Students noted a change in nurses/midwives' perception about them from being ‘a nurse/midwife help’, ‘two more hands’ or ‘not a smart girl’ to a perception of them as ‘an essential part of healthcare team’ in the DEU. Students expressed a shift in their self‐perception from being ‘not valuable’ in the TM to the feeling that they were ‘needed in the ward’ *in the DEU*. Students stated that, in the TM, they had to ask for nurses/midwives' permission to do things, and in some cases, students felt they were not accepted into the healthcare team. In contrast, in the DEU, students' experience was needed and appreciated by the staff, and they were supported by the healthcare team as new member who is just ‘starting a new placement’. Students' need to feel a sense of belonging during placement was a common finding across the five countries. Students valued being part of the healthcare team, feeling welcomed in the ward and considered they provided indispensable nursing care. A student added: ‘During the DEU placement, trust was definitely greater. I felt that they (mentors) wanted to help me, not judge me, that I would learn something from my theoretical classes’.

#### 3.3.4 Nurses/midwives' involvement in student learning

Students described an increase in everyone's awareness of their learning process. They also highlighted mentors and teachers' commitment to working together to involve the rest of the healthcare team in student education. Clinical staff stated that there was a better learning climate, and an increase in other professionals' awareness of student learning needs and in everyone's readiness to identify learning opportunities and in creating a ward’ ‘learning culture’. Students felt that more staff were willing to teach them. In contrast to the TM model, improved staff predisposition encouraged all other healthcare professionals to seek new student‐learning opportunities, while students believed that staff engagement in teaching not only depended on the learning model implemented but also on individual nurse/midwife characteristics. Thus, students asserted that some nurses/midwives are less willing to teach and that this affects their motivation and learning. A teacher stated: ‘…but in my case, the most relevant thing has been the motivation of the nurses, the involvement they have had in teaching… I have seen them super‐motivated…’.

### 3.4 Theme 4. Participants' suggestions for creating an effective learning environment

#### 3.4.1 Agreement on the learning–teaching process

Students suggested an agreement on learning objectives and expectations between university professors and clinical staff could reduce uncertainty. Students expressed the view that there were still discrepancies between nurses/midwives' set learning goals and emphasized the need to standardize teaching performance and learning objectives. Students stated that there was better follow‐up when nurses/midwives' schedules allowed students, teachers, mentors and head nurses to hold weekly meetings to plan and discuss student learning on the ward. A teacher stated: ‘For the final assessment we were all together‐teacher, mentor and head nurse‐ to share our experiences and vision on the student's progress’.

#### 3.4.2 Clinical placement early planning

In our study, all participants expressed a need for early placement planning. Nurses/midwives and students stressed the importance of the mentor having time to explain things, to demonstrate the practice skill and to wait for students to perform it without pressure. Staff highlighted the need for timely, realistic planning and scheduling before beginning student placements to provide clinical practice with appropriate mentor–teacher teams and with time to ensure a high‐quality teaching–learning process. A student stated: ‘Sometimes I felt that I arrived at the places and the nurses/midwives were not prepared to receive me… there is no team preparation for reception. There should be more coordination between university and service’.

#### 3.4.3 Investment in nurses/midwives

Some nursing/midwifery staff perceived working with a student as an increase in their workload. They spent a lot of time teaching, guiding and supervising students and it consumed valuable time in the current high nurse–patient ratio and high‐workload ward environment. They reported that teaching requires more human resources, reduction in nurse/midwife duties or rest periods between one student's placement and the next. Staff reported a lack of recognition, compensation and help in handling this additional role and a need to incentivize and facilitate nurses/midwives' participation to attract new nurses/midwives and to tackle tiredness among those already working. Students were aware that teaching students constantly without time to rest could exhaust nurses/midwives and that sometimes it decreased the quantity and quality of teaching time. A mentor stated: ‘Rest is also necessary. Investment in the mentor team, greater than it is now, is necessary to be able to continue and to make it bearable over time. We have to ensure that mentors do not become exhausted’.

## 4 DISCUSSION

Despite our heterogeneous sample with professionals and students of different ages and backgrounds, responders generally reported positive experiences in the DEU. Below are the elements highlighted by the participants that have improved with the implementation of the DEU and/or are considered essential to optimize the learning environment are discussed.

### 4.1 Clinical placement organization

As literature shows, good educational service collaboration brings a mutual understanding of student curricula, defines roles and responsibilities, promotes clinical education and evidence‐based practice and thus improves patient care (Huston et al., 2018). In our DEU, professionals' roles and responsibilities were clearly defined. While the mentor provided clinical knowledge and skills and acted as a role model, the teacher was responsible for guiding mentors in their mentoring role and ensuring that students were ‘fit for practice’. The ward ‘learning culture’ between staff, teachers and students was enhanced in our DEU. As the literature shows, good educational service collaboration brings a mutual understanding of student curricula, defines roles and responsibilities, promotes clinical education and evidence‐based practice and thus improves patient care (Huston et al., 2018). Regarding student placement duration, our findings show that longer placements increased students' learning opportunities, with time to develop practical skills and relational abilities with patients and other professionals. These targets are difficult to meet in short placement periods (Saukkoriipi et al., 2020). We also found that personalized mentor supervision focused on students' goals with mutual respect and approval promotes organization, collaboration and initiative in delivering care and has a good effect on clinical experience outcomes, as described in Perry et al. (2018) and in Pitkänen et al. (2018).

### 4.2 Students' clinical knowledge and skill acquisition

It is significant that the transfer of theoretical knowledge to clinical practice was difficult despite close collaboration between mentors and teachers. This gap was one of the major challenges for nurse and midwifery students in clinical environments (Panda et al., 2021). However, our participants recognized that the teacher helped to narrow these inconsistencies and our mentors and head nurses expressed their commitment to teaching students how theory and practice are integrated in direct patient care. Aligned with our results, research shows that to optimize a clinical learning environment, teachers and clinical nurses need to collaborate and be aware of clinical learning opportunities (Pedregosa et al., 2021). As in recent studies, this study indicates that the role of the teacher in the DEU has proven to be a good strategy to bridge the gap between theory and practice so that students learn to apply theoretical and practical principles flexibly to various clinical situations. Together with the nurse mentor, the teacher has the responsibility of linking the theory and research given during classes with clinical practice, care planning and decision‐making with the aim of achieving learning outcomes, promoting independence, responsibility and critical thinking of the student (Schoening et al., 2021; Shoghi et al., 2019).

### 4.3 Students and nurses/midwives within the DEU

Our findings are in line with previous studies showing that staff attitudes are a meaningful element for students to fit in the learning environment and influence students' self‐efficacy and preparation for practice (Panda et al., 2021). Our students appreciated the timely and non‐intimidatory manner in which staff tested their knowledge, supervised their work or offered constructive feedback. Moreover, our professionals showed more awareness of students' learning expectations than in the traditional model. Staff nurses with a professional mindset that values engagement, respect students as learners and provides them with a sense of belonging and confidence, influence student learning (Jack et al., 2018; Perry et al., 2018; Tomietto et al., 2016). Similarly, we found that student socialization and inclusion in the healthcare team were important for student well‐being, sense of belonging and effective clinical teaching. The literature shows that students' confidence in performing tasks and asking for help improves learning and that team efforts to involve all staff in building student abilities positively influence their attitude towards students (Lee et al., 2018). On the other hand, few studies have considered the role of the head nurse in clinical education. Frequently, the head nurse responsibilities consist of orienting students on the ward and encouraging staff to involve students. In line with Alammar et al. (2020), our students considered the head nurse as a team member who offered valuable input and treated them as a vital resource and enhanced their clinical learning. Head nurses' leadership style is fundamental in ensuring a high‐quality and safe learning environment due to their influence on staff motivation, work environment and learning climate and to stimulate students' interest in clinical practice (Zhang et al., 2022). Besides, our results are in accordance with Jansson and Ene (2016) and Pramila‐Savukoski et al. (2020), in that continuous feedback benefits the learning process and self‐reflection, maintaining student motivation and active involvement. Likewise, the absence of mentor feedback due to lack of time and workload leads to student dissatisfaction and has a significant impact on student self‐esteem and learning (Munawar et al., 2019). Furthermore, our students stated that a trusting relationship between mentor and student is essential for high‐quality instruction. As Perry et al. (2018) found in their study, the essential requirement for mentors was to be aware of students' knowledge, abilities and progress. Additionally, student autonomy and self‐confidence are related to supervisors' trust in their abilities, which also encourages learning (Perry et al., 2018). Jansson and Ene (2016) and Jack et al. (2018) showed that when the mentor is not aware of the students' clinical knowledge and abilities, or students are ignored or unsupported, they take responsibilities they are not prepared for, which puts patient well‐being at risk.

### 4.4 Participants' suggestions for creating an effective clinical learning environment

Our findings reinforce the idea that the learning environment should be carefully organized with a clear definition of each participant's role and responsibilities, realistic schedules and educational service managers commitment to ensure administrative and human resources are available to facilitate the experience (Rodríguez‐García et al., 2021; Sadeghnezhad et al., 2018). Our nurses/midwives highlighted their readiness and motivation as a benefit of the DEU preparatory training. In most European countries, there are no specific educational requirements or training strategies for nurse mentors (Dobrowolska et al., 2016; Tuomikoski et al., 2020). Furthermore, there is an unmet responsibility from academic and healthcare managers to evaluate the nurses' competencies, a lack of preparation to instruct students and provide them with knowledge, teaching–learning skills, professional growth opportunities and organizational support and considering their workloads when assigning students to them (Mikkonen et al., 2022). This derives from mentors having a feeling of isolation, anxiety and uncertainty, and a lack of preparation, recognition, time and support in the face of student difficulties or in vital aspects of their progress (Cusack et al., 2020; Quek & Shorey, 2018) when the adequate training for the role may increase perceptions of support and, as a result, commitment, increasing the preceptor's ability in the role (Macey et al., 2021). In this regard, in the current healthcare system in countries like Spain, Poland and Portugal, mentors do not have enough time to teach students due to their workload. These countries have a very high patient/nurse ratio. For instance, in Spain, a nurse takes care of about 13 patients on average (Edward et al., 2017). Heavy workloads were identified as the biggest issue for both mentors and teachers and there is pressure on staff to take on an educational role, with insufficient preparation and support due to demand in working conditions that are not ideal (Macey et al., 2021; Williams et al., 2021). Teaching is a very time‐consuming responsibility, with low recognition, and in some cases, it is not a priority concern of managers. Not responding to nurses' requests can compromise the quality and quantity of student supervision (Macey et al., 2021), and adequate training of students is a key factor in the provision of quality nursing care. Several authors have illustrated various components of what may contribute to DEU sustainability, including an economic evaluation for the clinical partner, nurse retention, orientation and training times, recruitment costs and nurse turnover rates. These authors observed that cost‐effectiveness and monetary savings were associated with the successful sustainability of the DEU (Pfannes, 2019).

## 5 CONCLUSIONS

Responders generally had positive experiences in the DEU and the required standards to create a DEU successful learning environment were highlighted: close educational service collaboration along with frequent and accurate feedback and trust in communication between faculty and staff; structural and human resources to construct sites where future nurses/midwives can develop personal and professional skills and attitudes to effectively deal with nursing challenges; all nurses/midwives' participation in the model implementation and process; and more clarity into professionals' roles and responsibilities. Furthermore, managers' contribution with the necessary support, time and skills to perform each role effectively and respond to educational needs through initiatives for continuous educational training were identified as successful learning outcomes in the clinical context. From educational and healthcare institutions, as professionals and inherent educators, we must adopt a positive attitude towards learning, establishing effective programs that improve the training and competence of students and professionals and optimize the clinical learning environment, in addition to the students and professionals learning experience, satisfaction, competence and professionalism.

## 6 STRENGTH AND LIMITATIONS

This is the first study which has evaluated the teaching–learning process within the DEU in different countries' clinical placements from the professionals and students perspective. Due to organizational and availability issues, DEU implementation was done in a few units per country, therefore, it is limited to a small number of participants. Despite staff being trained to manage qualified professionals' translations of participants' contributions, linguistic factors may be a limitation in a qualitative cross‐language study. The specific elements of the different curricula of educational institutions and the diverse healthcare systems participating in this study were not subjected to deep analysis. When carrying out future research, a necessary requirement would be to have more participants and include patients and the rest of the professionals who are part of the healthcare team due to their great impact on the students' clinical experience.

## AUTHOR CONTRIBUTIONS

AZ, SP: Research design. AZ, SP, NF, ER, MP, EW, FS, SM: Data collection, Analysis, Literature search, Manuscript preparation. All authors read and approved the final manuscript. All authors listed meet the authorship criteria according to the latest guidelines of the International Committee of Medical Journal Editors and all authors are in agreement with the manuscript.

## FUNDING INFORMATION

This research was supported by funding from the (1) European EPOS‐Erasmus+ program (IDEUs‐EU 2015‐1‐BE02‐KA202‐012329), (2) Fundació Catalunya‐La Pedrera (2018 Talent program) and (3) Barnaclínic (2018 sabbatical fellowship).

## CONFLICT OF INTEREST STATEMENT

None.

## ETHICS STATEMENT

The Ethics and Research ‘REDACTED’. Participant's personal information was collected by code to ensure confidentiality. Professionals and students were free to contribute to the study. If they choose not to participate, all the services they receive at these organizations will continue and it will not affect any aspect of their learning–working or related evaluations in any way.

## Supporting information


File S1.

## Data Availability

Data is available under request.
