# Supplementary material for: Nurses, midwives and students' reports of effective dedicated education units in five European countries: A qualitative study
Source: Nurs Open. 2024 Jul 3;11(7):e2210. doi: 10.1002/nop2.2210 (PMC11220640; doi:10.1002/nop2.2210)
Supplement: Supplementary file 1 — File S1. [file NOP2-11-e2210-s001.docx]

**Supplementary file 1. Students, nurses and midwives’ representative comments.**

| THEME 1. Clinical placement organization | |
| --- | --- |
| 1.1. Collaboration between educational and service institutions | **Student (s16):** *“(In DEU) There was a greater partnership between the service and the school and a better partnership between nurse and teacher”.*  **Clinical mentor (p15):** *“Collaboration between institutions was very good (in DEU); we all become part of the same team”.* |
| 1.2. Focus on student-learning process | **Student (s16):** *“The main difference (within DEU), is a greater follow-up. Weekly meeting (in DEU) allows everyone to follow the whole process better”.*  **Link teacher (p18):** *“When assessing the objectives weekly, it was possible to make a more continuous assessment”.*  **Student (s14):** *“…(in DEU) we didn’t have time to sit down and do nothing. There was simply no such time; we were working all the time. When there were no patients, the clinical mentor was saying: ‘Come on, I’ll tell you something about drugs’”.*  **Clinical mentor (p3):** *“…It makes the situation realistic; they feel that there are still opportunities to grow”* |
| 1.3. Longer placements enhances student autonomy and responsibility | **Student (s10):** *“…It takes 3 to 4 weeks to get to know the unit, to be oriented”. “(After this), I can understand better how patient care is organized and the tasks and the contributions of other caregivers”.*  **Clinical mentor (p4):** “*The student had more time to get into the work, to get introduced to patient care. They get more used to patient care and the unit/team. In (only) 3 weeks that was never possible for the students…it was not possible to give the student autonomy”.* |
| 1.4. One-to-one student to mentor is the best ratio for the teaching-learning process | **Student (s11):** *“That you can work mostly with the same nurse, who is a mentor and you don’t need to adjust to someone else. Not adapting every time…”.*  **Clinical mentor (p31):** *“One-to-one relationship was very important because we learnt the learning style of the students and we could develop our skills for students. We tried to find new ways to be specific for this person…before we didn’t because there were lot of students”.* |
| THEME 2. Students’ clinical knowledge and skill acquisition | |
| 2.1. From theory to practice | **Link teacher (p26):** “*The system is the same as when I studied. I dedicated many hours to issues that were very beautiful, very idealistic. But when you arrived at the unit you had to know how to administer atropine or adrenaline… many students did not know”.*  **Clinical mentor (p16):** *“LT should give theoretical support, protect didactic matters, watch over what student should learn according to the syllabus and mediate problems between student and mentor”.*  **Student (s18):** *“I am at the intensive care unit. In the HEI, we did subjects/themes about critical patients in only two weeks. And now, really, I do not know anything about these patients’ care”* |
| THEME 3. Student, nurses and midwives within the DEU model | |
| 3.1. Roles and responsibilities in the teaching-learning process. | **Head nurse (p13):** *“Thanks to individual approach to students and very clear distinction between clinical mentor and link teacher, both theoretical and practical education was much easier”*  **Link teacher (p18):** *“It made the role of all stakeholders more visible”.*  **Head nurse (p23):** *“…to achieve it (Clinical practice improvement), and go forward, previous training was fundamental. Nurses somehow feel involved in a project; training guidelines for trainers who have never had it, it seems basic”.*  **Clinical mentor (p28):** *“I have tried to apply what we have learned in DEU training. Because I thought that it was good”*  **Student (s16):** *“Presence of the link teacher made possible to integrate it (the theory into practice). She talked a lot with head nurses and clinical mentors, helped us to realize what we could do to have better experiences, and learning opportunities increased”.*  **Link teacher (p9):** *“The biggest advantage (in DEU) is that you are more part of the health team. If you are in the office, students can´t reach you easily… There are so many changes in nursing and if you are sitting in the office, then you don’t know about these changes. And if you are aware of all these things by still working in care, I think you can guide the student better as teachers (than in traditional model)”.*  **Link teacher (p26):** *“I know a lot about the surgery-intensive care unit. But if you send me to the cardiology unit, and if I have to teach students here and spend time with cardiac patients, I would not know how to explain things to them. The link teacher should also be a unit expert”.*  **Student (s33):** “*We respected each other and had very good communication. We weren’t afraid to ask anything; during the traditional placement it was very hard even to communicate with nurses/midwives but here we had a mentor and they were like friends but teaching, showing us the new ways”.*  **Clinical mentor (p15):** *“Mentor is not only a teacher. Mentor empathizes with the student and can see what bothers him/her. This is very important role. I was close to my student, so when I had to do something on my own, I was sad that the student couldn’t do it with me”.*  **Student (s16):** *“Greater involvement of the head nurse allows acquisition of more knowledge, to encourage this work among the nurses”*  **Head nurse (p23):** *“I participated in almost nothing before (in the traditional model). I have noticed a significant change in that sense. We do not have anything structured here, either. But I have felt more involved, more aware of the learning process”.* |
| 3.2. Participant interaction | **Student (s20):** *“HEI ask you for one thing, and here (on the ward) it is something different. Well, what I believe is that there is a lack of communication between HEI and hospital about clinical placement…”.*  **Clinical mentor (p10):** *“More communication between us and with the teacher. We discuss more than before. There (in the traditional model) were a lot of different teachers, they changed a lot and we did not know them well”.*  **Link teacher (s25):** *“We lacked this communication…I have found that students who came did not know where they were going to go”.*  **Student (s1):** “*Mentor was very consistent in making time for feedback. The quality of feedback was very good, because they were all so well informed and deeply involved. It gives the mid-period assessment a special, strong quality. It was very clear. I could really recognize myself in the feedback”.*  **Student (s4):** *“Further in the placement we got a lot of autonomy, then I sometimes I missed strong feedback”.*  **Student (s1):** *“…the mentor also talked about the student personal life…we can participate in the team as a close partner; there was also an opportunity to talk about myself as a person. They were really interested in my hobbies...”.*  **Clinical mentor (p19):** “*Realized better the investment that the student made. In the meetings, the student gave an opinion, what she did, what she felt, what difficulties she had. She showed more of what she was doing…It was an advantage for all parties involved”.*  **Head nurse (p29):** *“It was totally different. Students could bring new information to us. Because they were working with clinical mentor and it was very easy for them to give their opinion to the mentor”.*  **Student (s6):** *“We experienced a lot of trust in the collaboration with mentors, we got access to all information, and we validated medication under supervision… for us they are symbols of trust between us as a students and the mentor as a professional”.*  **Clinical mentor (p12):** “*You trust them more after time. Because you have a connection. If you have the student for a short time, you can do things but you don’t know what she can do, so you always go with the student”.* |
| 3.3. Students’ sense of belonging to the health team | **Student (s13):** *“During the DEU placement trust was definitely greater. I felt that they (mentor) wanted to help me, not judge me, that I would learn something from my theoretical classes”*  **Clinical mentor (p16):** *“Midwives wanted ‘to make use of’ the student, not to teach them: They said ‘bring this or that, clean those things”. Students shouldn’t be treated like ‘stupid girls’, who only help, but as a people who will become midwives soon and they will show good or bad behaviors…In the past, it was simple with a student. The nurse had an ‘extra pair of hands’”.*  **Student (s1):** *“Once they (others professionals) go to know us better, we became more part of the health team and they treated us more as close partners. They became friendly and we had more contact…they got curious about what we were doing and gave us more responsibility to do things”.*  **Head nurse (p8):** *“And everybody can say what they want. It is everybody’s right to say things. To say shouldn’t we do it like this or this, even students”.* |
| 3. 4. Nurses/midwives involvement in student learning | **Student (s19):** “*…not only the students ask, but also the staff, who are there, staff willingness. Staff say: ‘Hey! Do you have a minute? Because I want to teach you something”…They are more aware of educational part and the predisposition is very good”.*  **Head nurse (p17):** *“Whenever a need is detected by the team accompanying the process, the HN tries to fill in it and give support”.*  **Student (s19):** *“If you do not understand something you can ask any nurse. In traditional units, I have asked and they tell me: ‘ask another person, I am busy. Or ‘ask your nurse’ or ‘now I cannot answer you’. I have seen more willing staff (in DEU)”.*  **Student (s23):** *“…my nurse is not at all willing to teach me things. She is very nice and she is a very good nurse…but when she comes to doing things (she says): ‘let's do this technique, look at the protocol and if you have any questions, you can ask me’”.*  **Link Teacher (p25):** *“…but in my case, the most relevant thing has been the motivation of the nurses, the involvement they have had in teaching… I have seen them super-motivated, they made a check-list with the contents that they wanted the students to learn during practice”.* |
| THEME 4. Participants’ suggestions for creating an effective learning environment | |
| 4.1. Agreement on the learning-teaching process | **Student (s10):** *“In my opinion, my mentor had different expectations than her mentor (looks at another student) and maybe it should be more consistent”.*  **Link teacher (p2):** *“For the final assessment we were all together, teacher, mentor and head nurse to share our experiences and vision on the student’s progress. We prepared the final assessment together, I wrote down the assessment and feedback, everyone* *could reread the evaluation and give feedback. We discussed the qualification together”.* |
| 4.2. Clinical placement early planning | **Student (s15):** *“Sometimes I felt that I arrived at the places and the nurses/midwives are not prepared to receive me… there is no team preparation for reception. There should be more coordination between university and service”.*  **Head nurse (p1):** *“It is a noble goal to go for the triangle (teacher-mentor-student). But structural organization aspects such as holiday planning, sickness, workload, absent colleagues…often make it impossible to do. To make it possible we need to know many months in advance how many students will come for a placement.... And also, you need enough mentors”.*  **Link teacher (p27):** *“Meeting all together and do like what we've done in training courses...Try to create this learning culture, I don’t know, maybe through the training, to involve everybody. It is clear that mentor and teacher are mainly responsible for student training, but the rest are, with their eyes a little more open, if they collaborate at some point in explaining something...”.* |
| 4.3. Investment in nurses/midwives | **Student (s19):** *“But the best could be rotating nurses/midwives who teach students, and give them time to rest would also be fine… there are students who are having a very bad placement time and maybe it is because the nurse cannot take a rest from teaching students”.*  **Clinical mentor (p3):** *“Rest is also necessary. Investment in the mentor team, greater than it is now is necessary to be able to continue and to make it bearable over time. We have to ensure that mentors do not become exhausted”.* |

**DEU: Dedicated Education Units. HEI: Higher Education Institution**
